# Target activation and distractor inhibition on attentional bias in priming of popout search

**DOI:** 10.3758/s13414-025-03197-1

**Published:** 2025-11-21

**Authors:** Bryan R. Burnham

**Affiliations:** Department of Psychology, University of Scranton, Scranton, PA 18510 USA

**Keywords:** Feature priming, Selection history, Attentional bias, Activation, Inhibition

## Abstract

Selection history effects occur when visual search is facilitated after previous target features are repeated during subsequent searches relative to when target features switch with non-target distractor features. Selection history on visual search is likely due to a combination of feature activation (increased salience), bias in attentional decisions over target selection, and facilitated post-selection retrieval, and likely reflects both target activation and distractor suppression. The present study used a probe detection task within a standard priming of popout (PoP) visual search task to examine how target activation and distractor suppression influence attentional decisions to select a previous target’s features. PoP was observed in response times and importantly in recall of probes appearing on both color singleton targets and non-singleton distractors. Relative to baseline conditions, more probes were recalled from color singleton targets on color repeat trials, and fewer probes were recalled from targets on color switch trials; and more probes were recalled form the non-targets on switch trials than baseline trials. The results suggest that target activation and distractor suppression contribute to the attentional decision bias that arises due to selection history.

## Introduction

*Selection history* effects are a common finding in visual search studies, showing that the features of previously attended objects can prime the selection of those features during subsequent search (e.g., Awh et al., 2012; Chun & Jiang, 1998; Chun & Nakayama, 2000; Kristjánsson & Ásgeirsson, 2019, Kristjánsson & Campana, 2010; Lamy et al., 2008; Lamy et al., 2010; Maljkovic & Nakayama, 1994, 1996, 2000; Müller et al., 1995; Wolfe et al., 2003). For example, Maljkovic and Nakayama (1994) found that popout search for a feature singleton was faster when the target’s distinguishing feature was repeated between trials than when its feature switched with non-target features, even though its specific feature (e.g., red) was unpredictable. This classic *priming of popout* (PoP) effect – faster search when target-defining features repeat – has been attributed to several distinct mechanisms, including (a) feature-based enhancement (increased salience), (b) bias in attentional decision-making, and (c) facilitation of post-selection responses. Despite extensive investigation, these mechanisms often remain confounded in traditional search paradigms. The present study extends prior work by using a probe identification task (Gaspelin et al., 2015) within a popout search paradigm to isolate attentional bias, thereby separating it from post-selection retrieval and perceptual salience mechanisms.

Maljkovic and Nakayama (1994) presented a color singleton target among several homogeneously colored non-target distractors but randomly varied the target and non-target colors across trials. Although the specific target color was unpredictable, repeating the colors sped responding relative to when the target and distractor colors switched, suggesting feature repetition primed popout search. PoP has been observed using a variety of target defining features including color (Goolsby & Suzuki, 2001; Olivers & Humphreys, 2003), orientation (Hillstrom, 2000; Olivers & Humphreys, 2003), shape (Lamy et al., 2006), and size (Huang et al., 2004); and selection history effects have been observed during conjunctive search (e.g., Kristjánssona et al., 2002). While PoP is well documented, the present work contributes by isolating the contribution of attentional decision bias independent of perceptual salience or response priming. It does this by using a novel adaptation of the probe-letter task to evaluate attentional bias using probe recall and estimating the contributions of target activation and distractor suppression – two processes typically conflated in repeat versus switch comparisons.

Several processes have been proposed to account for selection history effects. According to pre-attentive (increased feature salience) accounts (e.g., Becker, 2008; Bichot & Schall, 2002; Kumada, 2001; Maljkovic & Nakayama, 1994, 1996, 2000), implicitly encoding a target’s features on Trial *N* – 1 boosts the activation of that feature on Trial *N*. Thus, selection history effects arise from “highlighting” the repeated feature through increased salience. For example, in macaque monkeys, Bichot and Schall (2002) found that activation in the frontal eye field – region associated with activations in a salience map – was greater on target repeat trials relative to switch trials.

Beyond salience-based explanations, other work suggests that selection history may also bias the decision of where to allocate attention without altering perceptual salience per se (e.g., Amunts et al. 2014; Burnham, 2023; Lleras et al., 2008; Tseng et al., 2014; Yashar & Lamy, 2010; Yashar et al., 2017). In diffusion-modeling terms, this reflects a shift in the starting point (z) of the decision process, which several studies have observed in PoP tasks (e.g., Burnham, 2018a, 2018b, 2023; Tseng et al. 2014); suggesting selection history influenced the decision over what to select. Others showed that PoP and the *distractor preview effect* occur only when selection of a target is required (e.g., Burnham 2018a; Lleras et al., 2008), suggesting it is not salience per se that leads to selection history effects, but active engagement with a target’s features.

It is also possible that selection history facilitates post-selection responding (e.g., Hillstrom, 2000; Huang et al., 2004; Huang & Pashler, 2005; Thomson & Milliken, 2011, 2013). Huang et al. (2004) found repetition of target features interacted with response repetition, suggesting selection history affected post-selection process. Thomson and Milliken (2011, 2013) observed that task changes across trials affected PoP, which suggests priming influenced response selection associated with each task. Despite evidence for this and each independent mechanism, many agree that selection history reflects multiple mechanisms (e.g., Ásgeirsson & Kristjánsson, 2011; Ásgeirsson et al., 2015; Becker et al., 2023; Burnham, 2018a, 2018b, 2023; Kristjánsson & Ásgeirsson, 2019, Kristjánsson & Campana, 2010; Lamy et al., 2010; Yashar et al., 2013).

One issue in PoP tasks is that by comparing *repeat trials* (target and non-target features repeat) to *switch trials* (target and non-targets features switch) there is no way to assess the independent contributions of activation arising from previous target selection and inhibition from ignoring previous non-targets (e.g., Kristjánsson & Driver, 2008; Lamy et al., 2010; Yashar & Lamy, 2011). That is, selection history may arise from activation from the preceding target feature, inhibition of the preceding non-target feature, or both (Kristjánsson & Driver, 2008). On repeat trials, responding may be faster because the current target’s feature was activated *and* the current non-target feature was inhibited, whereas on switch trials responding may be slowed because activation of the preceding target shifted attention toward the current non-target *and* inhibition of the preceding non-target feature slowed selection of the current target. In short, activation from the previous target and inhibition from the preceding non-target are confounded on repeat and switch transitions, so a baseline is important to assess contributions target activation and distractor inhibition.

## Present study

The present study aimed to quantify how selection history contributes to attentional decision bias by independently manipulating repetition and switching of target and distractor features – an approach that builds on but extends prior work (e.g., Lamy et al., 2008). In two experiments, in addition to repeat and switch trials, *baseline* trials were included in a standard popout search task. In Experiment 1, baseline trials were those on which neither the preceding target color nor non-target color carried over to the current trial. In Experiment 2, there were multiple baselines in which only the target color, only the non-target color, or both colors could repeat or switch between trials.

**Fig. 1:**
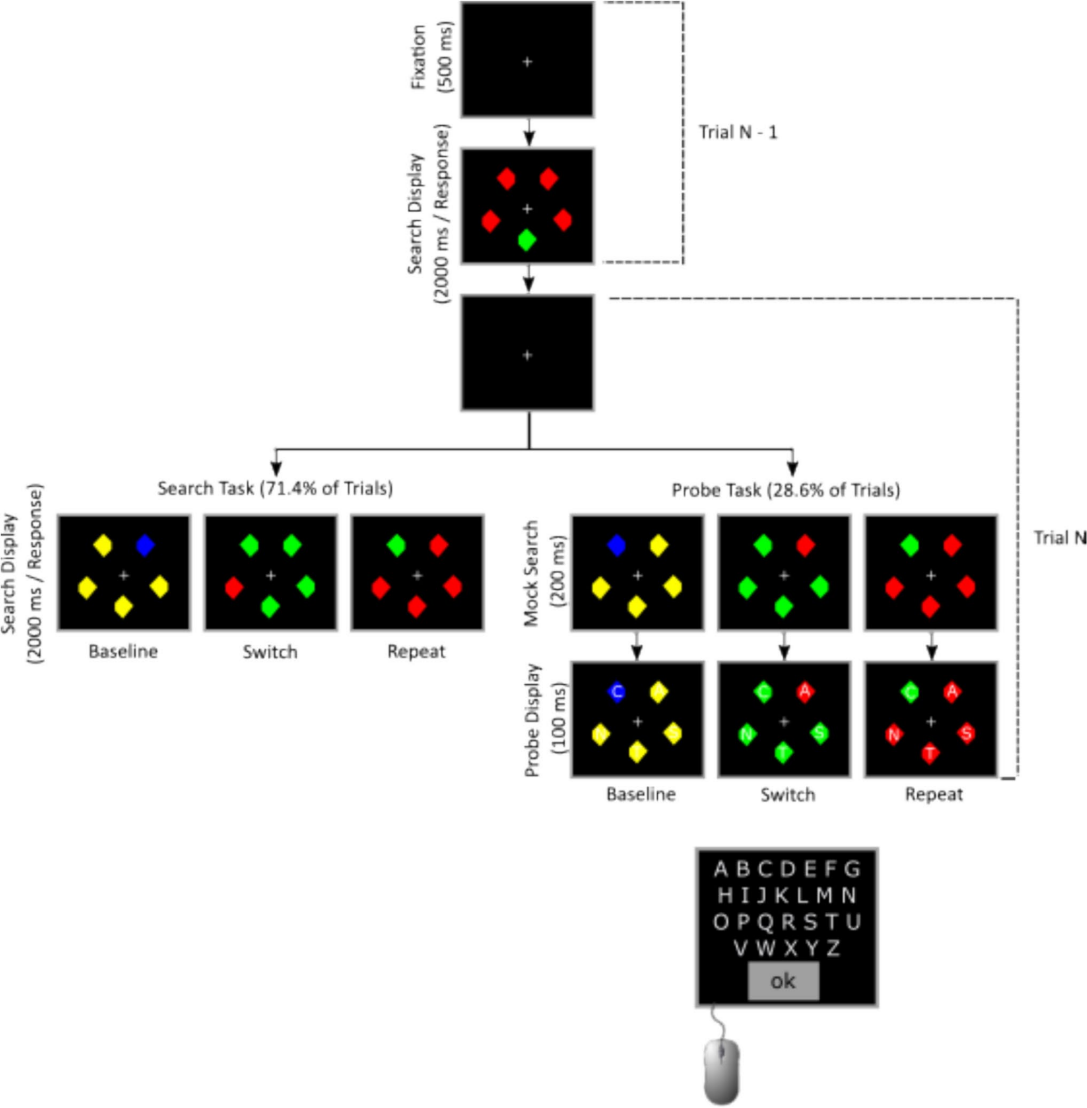
Trial sequence for the visual search and letter probe tasks. Stimuli are not to scale

In both experiments, in addition to the compound search task in which observers made speeded responses to which side of the target was missing (Fig. 1), *probe identification trials* were included on which observers identified letters that appeared on the target and non-targets. This probe task was adapted from Gaspelin et al. (2015), occurred on a minority of trials, and began exactly like the visual search trials. Specifically, a color singleton appeared among non-singletons that were identical in shape to the search items, with the colors repeated, switched, or new (baseline) from the preceding trial. However, after 200 ms, letters appeared briefly (100 ms) on each display item, which were then erased and replaced by a response screen that served as a mask. Observers selected as many letters as they recalled, with greater recall expected from those items on which attention was directed.

The probe task provided a way to examine attentional decision bias without influences from post-selection retrieval or increased salience. The brief (100 ms) presentation of letter probes, coupled with an immediate task switch, was designed to limit the influence of post-selection response-related processing (Huang et al., 2004; Huang & Pashler, 2005; Kerzel & Renaud, 2023; Lamy et al., 2008). Specifically, rapid onset and orthogonal response (letter identification rather than shape discrimination) should isolate attentional allocation from decision- or motor-level biases. Crucially, because selection history should not increase the salience of homogenous distractors, differences in probe recall from these items serve as evidence of bias in selection, not bottom-up capture. That is, if target activation biased decisions to select the preceding target color, recall of letters appearing on the non-targets should be *greater* on switch trials than on baseline trials, with little difference in letter recall from non-targets between repeat and baseline trials. Also, recall of the probe letters from the color singleton should be *lower* in the switch condition if the decision bias was affected by inhibition of the most recent non-target color. In sum the letter probe task allowed examination of target activation and distractor inhibition on attentional decision bias.

## Experiment 1

A popout search task occurred on most trials that was randomly interspersed with a letter probe identification task. On each trial, five diamonds appeared. One was a color singleton target and the other four were non-singleton distractors. On most trials, subjects identified whether the target was missing its left or right corner (search task; Fig. 1), and on other trials uppercase letters from the English alphabet appeared on each diamond and subjects identified as many as they could remember (letter probe task). Color-repetition effects and switching effects were expected to occur relative to the baseline condition in response times (RTs). For letter probe identification, if priming biased the attentional decision to select the most recent target color (target activation) and ignore the most recent non-target color (distractor inhibition), recall of letters on the color singleton should be lower on switch trials relative to baseline. Additionally, recall of letters appearing on the non-singleton distractors should be higher in the switch condition than in the baseline, as attention would be biased to select the preceding target color which now defined the non-singletons.

### Methods

#### Subjects

Previous studies in my lab produced large PoP effects (*d*_*z*_ > 1). In the present study, with three transitions (repeat, baseline, switch), a power analysis using GPower (Faul et al., 2007) indicated that at least 12 subjects were needed to detect a large effect (*f* =.25) to achieve Power =.90 (α =.05). *N* = 17 University of Scranton undergraduates signed up and participated (11 female; one left-handed). Subjects ranged in age from 18 to 23 years (*M* = 18.82, *SD* = 1.33 years) and reported normal or corrected-to-normal vision. All subjects passed an Ishihra colorblindness test.

#### Apparatus

The experiment was programmed and presented using (E-Prime) (2.0.10242) on a Dell 755 computer (1.96 GB RAM at 2.33 GHz). Subjects sat 60 cm from a Dell E178Fpv monitor with a resolution of 1,024 x 768 running at 60 Hz. A five-button response box was used for responding in the search task, and the computer mouse was used in the letter probe task.

#### Stimuli

Search displays contained five diamonds (1.1° x 1.1°) appearing on a black background (0.16 cd/m^2^, RGB: 0, 0, 0), and each diamond was missing its left or right corner (0.14°). One diamond was a color singleton target and the other four were homogeneously colored non-targets (see Fig. 1).^1^ The colors of the target and non-targets were chosen randomly on each trial to be red (20.44 cd/m^2^, RGB: 255, 0, 0), green (20.62 cd/m^2^, RGB: 10, 177, 31), blue (19.07 cd/m^2^, RGB: 91, 123, 253), or yellow (21.84 cd/m^2^, RGB: 142, 133, 15). To provide a single baseline for the repeat and switch transitions, red and green diamonds appeared together, *or* blue and yellow diamonds appeared together; there were no other color combinations (e.g., red with blue). A white cross (25.77 cd/m^2^, RGB: 255, 255, 255) appeared throughout each trial to maintain fixation. Each diamond appeared at a different one of 12 locations on the circumference of an imaginary ellipse (10° wide x 8° high) centered on the screen. Locations were selected randomly, but the target’s location was never repeated between trials.

The letter probe display consisted of five white letters in Sans Serif font (roughly 0.5° × 0.5°) that were chosen randomly and without replacement from the English alphabet, with one letter superimposed on each of the five diamonds in the display. The letter probe response display contained all 26 letters of the English alphabet and an “OK” key. The probe response display also served as a mask for the probe display.

#### Procedures

After providing consent, subjects were administered an Ishihara color blindness test, which all subjects passed. The monitor was then adjusted so a fixation point was at the subject’s eye level. Subjects received oral instructions that were also presented on the monitor. They were informed that most trials would be a visual search task to locate an oddly colored target diamond, but on some trials their task was to identify white letters that would appear on the diamonds. Subjects were informed the diamond colors could be red, green, blue, or yellow, but were not informed about the color pairings. Subjects were informed of the likelihoods of each task within a block, but the task on any one trial was randomly selected.

For both tasks (Fig. 1), subjects were informed they would see five diamonds, one of which was a different color. For the search task, they were told to indicate whether the target’s left or right corner was missing as quickly and accurately as possible. Subjects pressed the left key on the response box if the left corner was missing and the right key if the right corner was missing. For the letter probe task, subjects were instructed to not respond to the missing corner on the color singleton, but to remember as many letters as possible. They then used the computer mouse to select between zero and five letters and clicked “OK” when they could not recall more letters.

All trials began with a fixation display for 500 ms, followed by a display with five diamonds, so both the search task and letter probe task began the same. For the search task (71.4% of trials in a block), the diamonds remained visible for up to 2,000 ms or until a response was made. If a subject responded incorrectly or 2,000 ms passed, a 100-ms 500-Hz tone was played. For the letter probe task (28.6% of trials in a block), the five diamonds appeared for 200 ms and white letters then appeared (one per diamond) for 100 ms. The diamonds and letters were erased, and the probe response display was presented and remained visible until the subject selected “OK” to begin the next trial. Participants were not given feedback about letter recall accuracy.

Participants first completed a practice block of 48 trials with just the visual search task, followed by a second practice block with 32 visual search task trials and 16 letter probe trials. Subjects then completed 10 blocks of 112 trials each (80 visual search and 32 letter probe). At the end of each block, subjects were encouraged to rest their eyes before continuing.

### Results

**Fig. 2:**
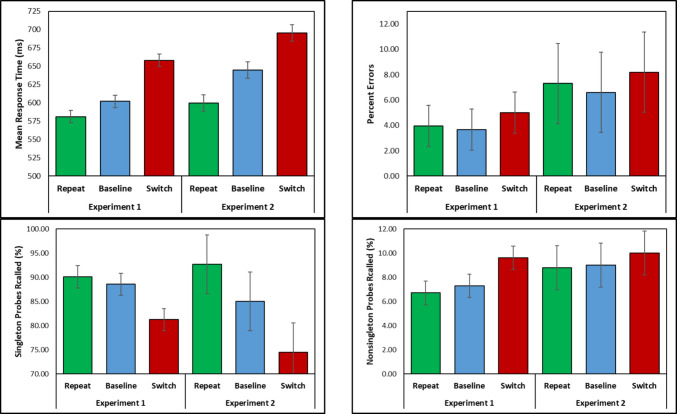
Mean response times (RTs), percent errors, and percentage of color singleton and non-singleton letter probes recalled in the repeat, switch, and baseline conditions Experiments 1 and 2. Error bars are the 95% confidence intervals

Three between-trial transitions were created based on the colors present in the current trial (Trial *N*) and preceding trial (Trial *N* – 1): (1) *Repeat*: target and distractor colors repeated. (2) *Switch*: target color switched with the distractor color. (3) *Baseline*: Trial *N* did not contain colors from Trial *N* – 1. For all analyses (response times (RTs), errors, probe responses), performance was assessed on Trial *N* only when a search task occurred on Trial *N* – 1. This was done so carryover (priming) onto Trial *N* would be equal for the search task and probe task, and so that a response mode change from an observer moving their hand from the mouse to the keyboard did not affect performance on the search task. For RT analyses, only trials with a correct response on both Trial *N* and Trial *N* – 1 were used, which resulted in the removal of 7.61% of eligible trials. For error analyses, only trials with a correct response on Trial *N* – 1 were used. For the letter probe analyses, only trials with a correct response on Trial *N* – 1 were used, which resulted in the removal of 3.83% of eligible trials. Percentages of correctly recalled letter probes were calculated based on whether the recalled letter appeared on the color singleton target or a non-target (averaged across all four non-targets). Each subject’s mean RT (*M*_RT_), percent error, and percentages of correctly recalled letters appear in Table 1 and in Fig. 2.

**Table 1:** Mean response times (RTs), SDs, percent errors, and percent of letter probes correctly recalled on color singletons and non-singleton distractors for the color switch, color repeat, and baseline between trial transitions

Experiment	*n*	Transition	Search task			Letter probe task	
			*M*_RT_	*SD*	Errors	Singleton	Non-singleton
1	17	Switch	658 ms	65	5.01%	81.24%	9.62%
		Baseline	602 ms	51	3.65%	88.56%	7.30%
		Repeat	581 ms	50	3.95%	90.13%	6.71%
2	14	Switch	596 ms	95	8.17%	74.51%	10.03%
		Baseline	645 ms	99	6.59%	84.99%	8.95%
		Repeat	600 ms	79	7.34%	92.72%	8.82%

#### Response times

A one-way repeated-measures ANOVA with three levels (*Repeat, Switch, Baseline*) revealed a significant effect of transition [*F*(2,32) = 99.42, *MSE* = 271.22, *p* <.001, η_p_^2^ =.86]. To examine target activation from color repetition and inhibition from color switching, all three pairwise comparisons were made (*p*-values are Holm-corrected). Responding on repeat trials was 21 ms faster than the baseline trials [*t*(16) = 5.96, *SE* = 3.52, *p* <.001, *d*_*avg*_ = 0.38], responding on the switch trials was 56 ms slower than the baseline trials [*t*(16) = 10.33, *SE* = 5.43, *p* <.001 *d*_*avg*_ = 1.01], and the PoP effect (switch – repeat = 77 ms) was also significant [*t*(16) = 10.50, *SE* = 7.34, *p* <.001, *d*_*avg*_ = 1.33].

#### Errors

A similar ANOVA revealed an effect of transition [*F*(2,32) = 3.40, *MSE* =.002, *p* =.046, η_p_^2^ =.18]. Responding in the repeat condition was nearly as accurate as baseline [*t* < 1, *d*_*avg*_ = 0.10] and 1.4% more errors were made on switch trials than on baseline trials [*t*(16) = 2.18, *SE* = 0.006, *p* =.133, *d*_*avg*_ =.43], and the PoP effect of 1.1% was not significant [*t*(16) = 1.67, *SE* = 0.006, *p* =.228, *d*_*avg*_ =.33].

#### Probes

For probes that appeared on the color singleton, a one-way ANOVA revealed an effect of transition [*F*(2,32) = 19.12, *MSE* = 271.22, *p* <.001, η_p_^2^ =.86]. Though not statistically significant, 1.6% more probes were recalled when the colors repeated compared to the baseline trials [*t*(16) = 1.02, *SE* = 0.015, *p* =.321, *d*_*avg*_ =.22] and as predicted by attentional decision bias, 7.3% fewer probes were recalled when the colors switched compared to the baseline [*t*(16) = 5.76, *SE* = 0.013, *p* <.001, *d*_*avg*_ = 1.02]. The PoP effect of 8.9% was also significant [*t*(16) =5.04, *SE* = 1.76, *p* <.001, *d*_*avg*_ = 1.24].

For probes appearing on the non-singletons, the effect of transition was present [*F*(2,32) = 11.22, *MSE* = 0.0004, *p* <.001, η_p_^2^ =.41]. Though not statistically significant, 0.6% fewer probes were recalled when the colors repeated compared to the baseline condition [*t*(16) = 1.36, *SE* = 0.004, *p* =.194, *d*_*avg*_ =.08] and, importantly, 2.3% *more* probes were recalled when the colors switched compared to the baseline [*t*(16) = 3.856, *SE* = 0.006, *p* =.004, *d*_*avg*_ =.33]. The “reverse” PoP effect of 2.9% *fewer* letters recalled from non-singleton distractors was also significant [*t*(16) = 3.45, *SE* =.844, *p* =.007, *d*_*avg*_ = 0.41].

### Discussion

Color repetition facilitated responding to an upcoming target while color switching impeded responding, with priming effects observed in RTs and recall of letter probes. Importantly, recall of letter probes on the color singleton was *lower* following color switches, reflecting reduced attentional allocation to the current target when it shared features with the previously inhibited distractor color. Perhaps the most interesting result was that the recall of letter probes on non-targets was greater following color switches than color repetitions. This “reversed PoP” reflects a carryover attentional bias from the previously selected target color, which guided attention toward the non-singleton non-targets. The results also suggest that distractor inhibition from the preceding trial biased selection away from the current color singleton target, as recall of letter probes from the color singleton was lower on switch trials relative to baseline. Hence, both target activation and distractor inhibition influenced attentional decisions. These findings support the view that PoP effects involve lingering cognitive biases from prior selection episodes.

## Experiment 2

Experiment 2 was largely a replication of Experiment 1, but any combination of red, green, blue, and yellow diamonds was possible for the target and distractors in each display. Hence, between trials only the target color, only the distractor color, or both colors repeated, switched, or were new. This multiple baseline approach was adapted from Lamy et al. (2008; see also, Yashar & Lamy, 2010), who examined contributions of target activation and distractor suppression on PoP. Lamy et al. (2008) presented targets and non-targets in any of four colors and found that target activation and distractor suppression independently (additively) contributed to PoP; that is, neither repeating or switching the target color interacted with repeating or switching the non-target color.

### Methods

Unless noted, all methods were the same as in Experiment 1.

#### Subjects

A total of *N* = 14 University of Scranton undergraduates participated (eight female; three left-handed). Subjects ranged in age from 18 to 22 years (*M* = 18.73, *SD* = 1.27) and reported normal or corrected-to-normal vision and passed a colorblindness test.

#### Stimuli

All color combinations of the singleton target and non-singleton distractors were possible in any given search display (red-green, red-yellow, red-blue, green-yellow, green-blue, yellow-blue). Colors for the target and distractors were randomly chosen across trials, so the target color was unpredictable.

#### Procedures

In Experiment 2, participants completed a practice block of 48 trials with just the visual search task, followed by two practice blocks with 54 visual search task trials and 18 letter probe trials. Subjects then completed eight blocks of 128 trials each (96 visual search and 32 letter probe) for the study.

### Results

**Fig. 3:**
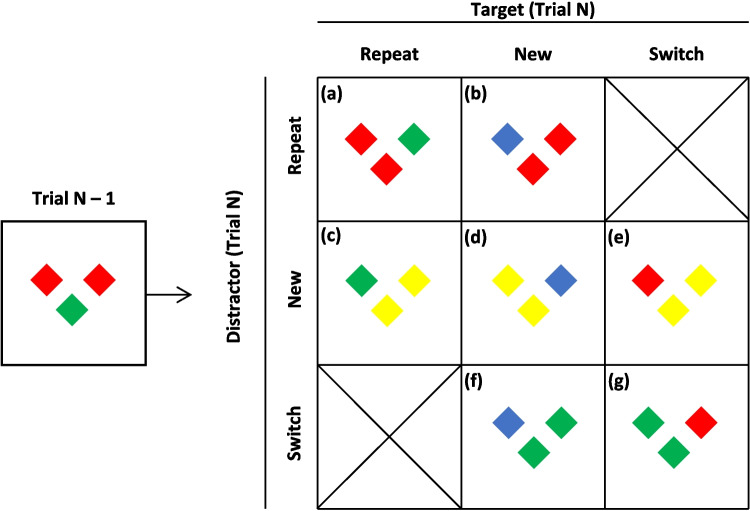
Example of the between trial transitions in Experiment 2: (**a**) *repeat: target and distractor repeat*, (**b**) *target new, distractor repeat*, (**c**) *target repeat, distractor new*, (**d**) *baseline*, (**e**) *target switch, distractor new*, (**f**) *target new, distractor switch*, (**g**) *switch: target and distractor switch*

**Fig. 4:**
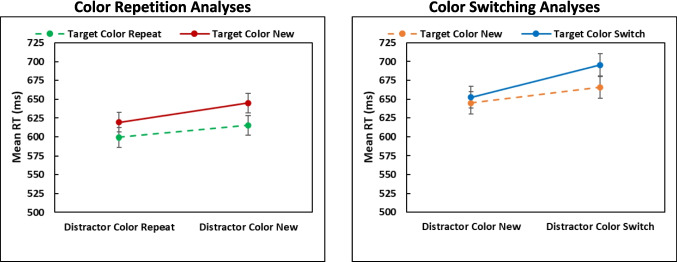

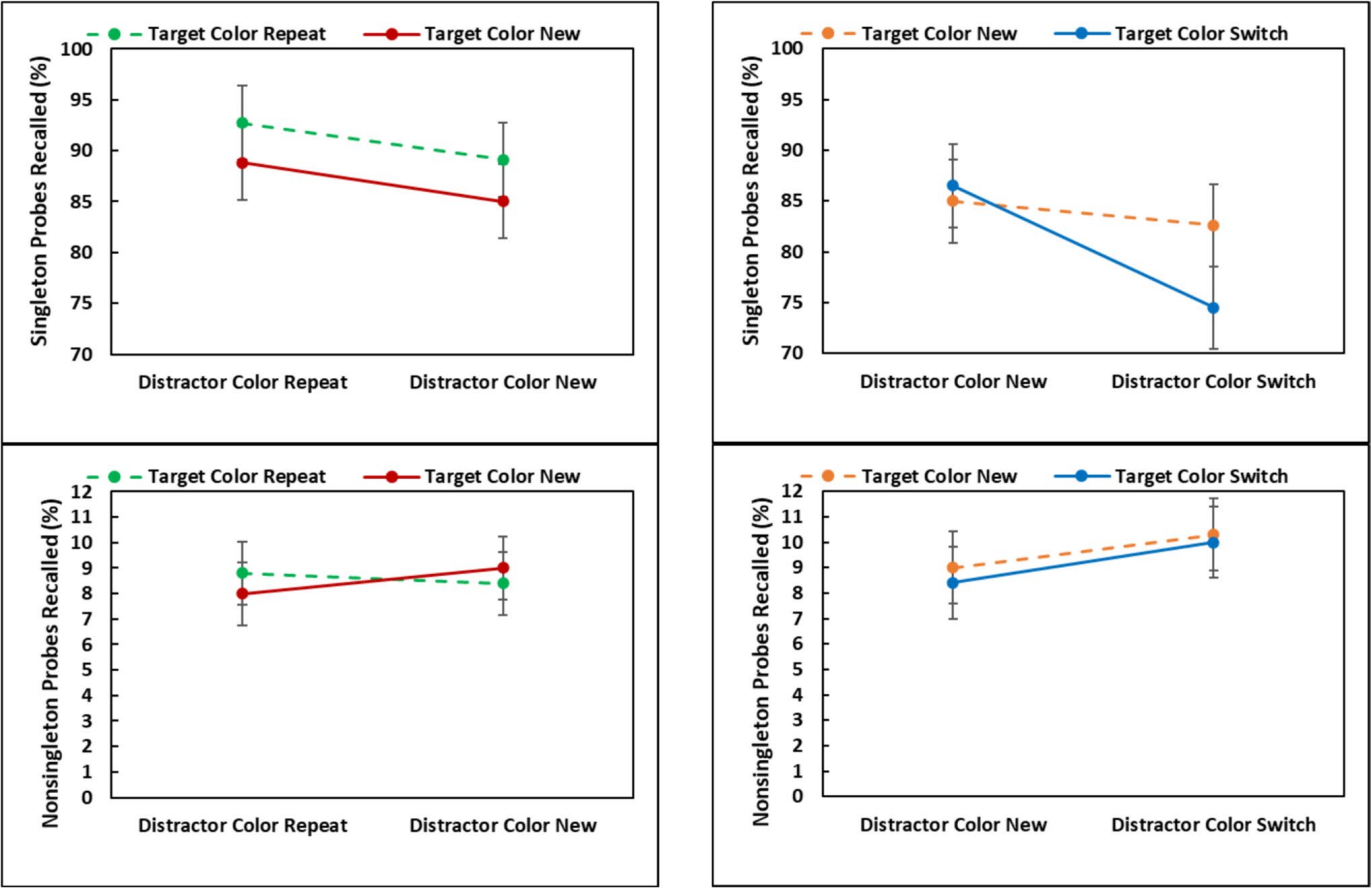
Mean response times (RTs) (upper panels) and percentage of letter probes recalled from the color singleton (middle panels) and non-singletons (lower panels) recalled in the 2 x 2 color repetition and color switching analyses from Experiment 2. Error bars are the 95% confidence intervals

On any Trial *N*, the target’s color (1) repeated from Trial *N* – 1, (2) switched from the distractor color on Trial *N* – 1, or (3) was not present on Trial *N* – 1. Similarly, the distractor color on Trial *N* (1) repeated from Trial *N* – 1, (2) switched from the target color on Trial *N* – 1, or (3) was not present on Trial *N* – 1. The three transitions for target color and for distractor color resulted in seven conditions (Fig. 3), as two conditions (target switch/distractor repeat; distractor switch/target repeat) were impossible. As the data could not be analyzed as a 3 x 3 factorial, separate 2 (Target: Repeat vs. New) x 2 (Distractor: Repeat vs. New) repeated-measures ANOVAs examined color-repetition effects, and 2 (Target: Switch vs. New) x 2 (Distractor: Switch vs. New) ANOVAs examined color switching effects. Statistical results from the search task and the letter probe task appear in Tables 2 and 3, and results are graphed in Fig. 4. Following these ANOVAs, estimates of target activation and distractor inhibition costs and benefits were derived as in Lamy et al. (2008), and compared to the overall PoP effects (switch – repeat). As in Experiment 1 the repeat, switch, and baseline conditions were first compared in separate one-way repeated-measures ANOVAs.

**Table 2:** Color repetition analyses: Results from 2 Distractor (Repeat, New) × 2 Target (Repeat, New) repeated-measures ANOVAs on the mean response times (RTs), percent errors, and letter probe performance in Experiment 2

Measure	Effect	*F*_(1,13)_	*MSE*	*p*	η^2^_P_
RTs	Distractor (Repeat, New)	19.41	311.49	<.001	.60
	Target (Repeat, New)	19.41	435.72	<.001	.60
	Distractor x Target	0.66	506.52	.432	.05
Errors	Distractor (Repeat, New)	0.01	.002	.917	.00
	Target (Repeat, New)	0.98	.0006	.341	.07
	Distractor x Target	1.32	.001	.271	.09
Probes-Singleton	Distractor (Repeat, New)	14.94	.001	.002	.54
	Target (Repeat, New)	4.59	.005	.052	.26
	Distractor x Target	0.00	.004	.958	.00
Probes-Non-singleton	Distractor (Repeat, New)	0.18	.0006	.681	.01
	Target (Repeat, New)	0.05	.0005	.827	.00
	Distractor x Target	1.53	.0004	.237	.11

**Table 3:** Color-switching analyses: Results from 2 Distractor (New, Switch) × 2 Target (New, Switch) repeated-measures ANOVAs on the mean response times (RTs), percent errors, and letter probe performance in Experiment 2

**Measure**	**Effect**	***F***_**(1,13)**_	***MSE***	***p***	**η**^**2**^_**P**_
RTs	Distractor (New, Switch)	72.29	197.77	<.001	.85
	Target (New, Switch)	19.93	246.32	<.001	.60
	Distractor x Target	2.66	646.22	.127	.17
Errors	Distractor (New, Switch)	0.28	.003	.604	.02
	Target (New, Switch)	0.37	.002	.554	.03
	Distractor x Target	0.51	.003	.489	.04
Probes-Singleton	Distractor (New, Switch)	9.54	.008	.009	.42
	Target (New, Switch)	2.10	.007	.171	.14
	Distractor x Target	6.46	.005	.025	.33
Probes-Non-singleton	Distractor (New, Switch)	2.85	.001	.115	.18
	Target (New, Switch)	0.21	.001	.654	.02
	Distractor x Target	0.04	.0006	.845	.00

Results from each 2 x 2 ANOVA were associated with *df*_Effect_ = 1 and *df*_Error_ = 13, with *N* = 14

Results from each 2 x 2 ANOVA were associated with *df*_Effect_ = 1 and *df*_Error_ = 13, with *N* = 14

#### Response times

**Repeat versus baseline versus switch.** A one-way ANOVA comparing the repeat, switch, and baseline transitions revealed a significant effect of transition [*F*(2,26) = 83.77, *MSE* = 385.80, *p* <.001, η_p_^2^ =.86]. Responding on repeat trials was 45 ms faster than on baseline trials [*t*(13) = 5.15, *SE* = 8.80, *p* <.001, *d*_*avg*_ = 0.50], responding on switch trials was 51 ms slower than on the baseline trials [*t*(13) = 9.46, *SE* = 5.35, *p* <.001 *d*_*avg*_ =.56], and the PoP effect was significant [96 ms; *t*(13) = 12.48, *SE* = 7.70, *p* <.001, *d*_*avg*_ = 1.05].

**Target activation and distractor inhibition**. The color repetition ANOVA yielded significant effects of target color repetition and distractor color repetition (*p*s <.001; see Table 2), but no interaction. Responding was faster when the target color was repeated (607 ms) than when it was new (632 ms), and when the distractor color was repeated (609 ms) than when it was new (630 ms). Similarly, the color-switching ANOVA (Table 3) produced effects of target switching (Trial *N* target color was Trial *N* – 1 distractor color) and distractor switching (Trial *N* distractor color was Trial *N* – 1 target color), but no interaction (*p* =.127). Responding was slower when the current target was the previous distractor color (674 ms) than a new color (655 ms) and responding was slower when the current distractors were the previous target color (680 ms) than a new color (649 ms). This slowing suggests residual inhibition of the distractor color from the prior trial, even when it becomes the target.

**Fig. 5:**
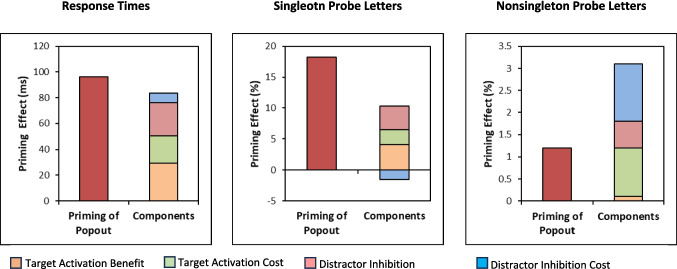
Priming of popout (PoP) effects and the summed components resulting from target activation and distractor inhibition in Experiment 2

To explore the influences of target activation and distractor inhibition, costs and benefits associated with selecting the target color and ignoring the distractor color were computed as in Lamy et al. (2008), by comparing a specific intertrial transition to baseline. The mean effects (differences), 95% confidence intervals (CIs), and effect sizes of each component appear in Table 4. The sum of the four RT components (84 ms; Fig. 5) was not significantly different from the PoP effect of 96 ms [*t*(13) = 1.42, *SE* = 8.74, *p* =.18, *d* =.38].

**Table 4:** Target activation and distractor inhibition benefits and costs from Experiment 2

**Component**	**Comparison**	**Measure**	**Effect**	**95% CI**	***D***
Target activation Benefit	Target repeat, distractor new vs. all new	RT	29 ms	[39,62]	.82
		Probe-Target	4.1%	[0,8.4]	.56
		Probe-Distractors	0.6%	[−0.9,2.1]	.22
Target activation Cost	Target new, distractor switch^a^ vs. all new	RT	21 ms	[1,41]	.60
		Probe-Target	2.4%	[−4.2,9.1]	.21
		Probe-Distractors	1.3%	[0,2.8]	.54
Distractor inhibition Benefit	Target new, distractor repeat vs. all new	RT	26 ms	[8,43]	.83
		Probe-Target	3.8%	[0,7.8]	.55
		Probe-Distractors	1.0%	[−0.4,2.4]	.40
Distractor inhibition Cost	Target switch^b^, distractor new vs. all new	RT	8 ms	[−3,18]	.40
		Probe-Target	−1.5%	[−6.2,3.1]	.19
		Probe-Distractors	−0.5%	[−2.1,1.1]	.18

See Fig. 3 for examples of the intertrial transitions that contributed to these components

^a^Distractor switch transitions occurred when the target color on Trial *N* – 1 became the distractor color on Trial *N*

^b^Target switch transitions occurred when the distractor color on Trial *N* – 1 became the target color on Trial *N*

#### Errors

As seen in Tables 2 and 3, the ANOVAs on the errors produced no statistically significant effects, and the one-way ANOVA that compared the repeat, switch, and baseline conditions revealed no effect of transition [*F* < 1].

#### Probes-color singleton

**Repeat versus baseline versus switch.** The one-way ANOVA comparing the repeat, switch, and baseline transitions yielded a significant influence of transition [*F*(2,26) = 11.03, *MSE* = 0.011, *p* <.001, η_p_^2^ =.46]. Overall, 7.7% more probes were recalled when the colors repeated compared to the baseline condition [*t*(13) = 3.19, *SE* = 0.024, *p* =.014, *d*_*avg*_ =.57], and 10.5% fewer probes were recalled when the colors switched compared to the baseline condition [*t*(13) = 2.74, *SE* = 0.038, *p* =.017, *d*_*avg*_ =.77]. The overall PoP effect was also significant [18.2%; *t*(13) = 3.65, *SE* = 0.050, *p* =.009, *d*_*avg*_ = 1.33].

**Target activation and distractor inhibition**. For probes appearing on the color singleton, the ANOVA examining color repetition yielded a significant effect of distractor repetition (*p* =.002) and a near-significant effect of target repetition (*p* =.052), but no interaction (*p* =.958). More probes were recalled on the color singleton when the target color was repeated (90.9%) than when it was new (86.9%), and when the distractor color was repeated than when it was new (90.8% vs. 87.0). The color-switching ANOVA yielded an effect of distractor color switching (*p* =.009), which surprisingly interacted with target color switching (Fig. 4, middle right panel). This interaction suggests that probe recall is particularly disrupted when both color roles switch, possibly due to episodic binding effects (cf. Giesen & Rothermund, 2014). Fewer probes were recalled on the color singleton when the distractor color was the Trial *N* – 1 target color (78.5%) than a new color (85.8%), which was pronounced when a full switch of colors occurred between Trial *N* and Trial *N* – 1. However, this did not occur in the RTs or recall of distractor probes. The sum of the four components of target activation and distractor inhibition (8.8%; see Fig. 5) was less than, but not significantly, the overall PoP effect of 18.2% [*t*(13) = 2.051, *SE* =.046, *p* =.061, *d* =.55.

#### Probes–non-singletons

**Repeat versus baseline versus switch.** Unlike Experiment 1, the ANOVA revealed no significant difference across the repeat, switch, and baseline transitions [*F* < 1], though the pattern of results was similar to Experiment 1.

**Target activation and distractor inhibition**. The ANOVA on color repetition did not yield any significant results (*p*s >.237), nor did the ANOVA on color switching (*p*s >.115). The sum of the four components of target activation and distractor inhibition components (2.4%; see Fig. 5) was not significantly different from the overall PoP effect of 1.2% [*t* < 1, *p* =.572, *d* =.16].

## Discussion

Comparison of the repeat, baseline, and switch conditions produced the same patterns as Experiment 1 and suggests that selection history biased attentional decisions through both target activation and distractor suppression. The purpose of Experiment 2 was to closely examine how target activation and distractor inhibition contribute to attentional decision bias by orthogonally manipulating target color and distractor color transitions. The results showed that both target activation and distractor inhibition contributed to PoP in a mostly additive manner (Figs. 4 and 5). This additive pattern aligns with prior findings (e.g., Lamy et al., 2008) but extends them by showing that decision bias – indexed by probe recall – follows similar dynamics. As seen in the rightmost panel of Fig. 5, target activation cost and distractor inhibition cost contributed most to priming for probes recalled from non-targets. The influence of target activation cost is not surprising, given that selection the Trial *N* – 1 target would bias selection of the same color on Trial *N*. The contribution of distractor inhibition cost suggests that ignoring (suppressing) the Trial *N* – 1 non-target color biased attention away from previously suppressed features, limiting their influence even when they reappeared as targets.

## Influence of color singleton shape/response

An assumption made in this study was that priming effects on probe recall were not due to episodic retrieval and reflected attentional bias. This was based on the quick change from a “mock” search display to the probe display and that a task switch occurred between a search trial and a probe trial. Even though probe responses (letter identifications) should not activate a preceding search target response (missing side), the *shape* of the color singleton on probe trials could be the same or different from the color singleton target’s shape on each preceding search trial. For example, a color singleton target on a Trial *N* – 1 search trial may be missing its left corner, and shape of the color singleton a Trial *N* probe trial may also be missing its left corner (or missing its right corner). As such, it is possible that when the shape (and associated response) of the color singleton on a probe trial matched the shape of a color singleton target on a preceding search trial, recall was better than when the shapes differed. Such a result would suggest episodic retrieval contributed to priming on probe recall.^2^

**Fig. 6:**
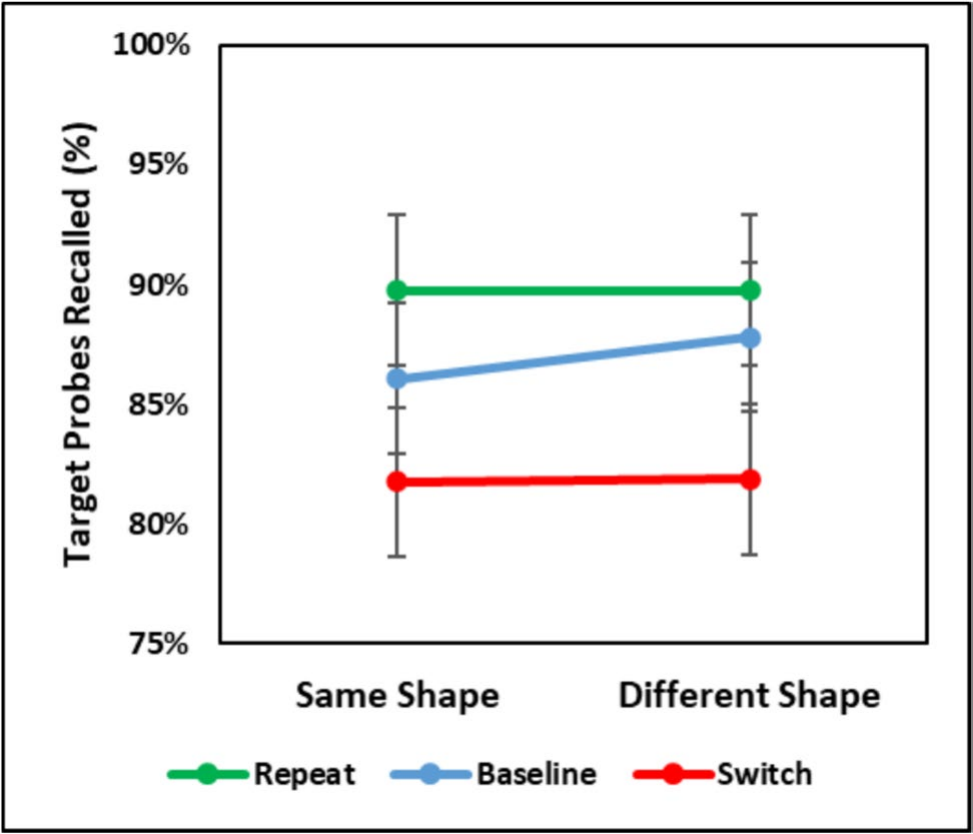
Mean recall of letter probes from color singletons in the combined data from Experiments 1 and 2 for the repeat, baseline, and switch transitions as a function of singleton target shape (same vs. different)

To examine this, recall of letter probes from the color singleton was assessed in the repeat, baseline, and switch transitions based on whether the missing side of the color singleton on Trial *N* – 1 and Trial *N* matched (*shape same*) or mismatched (*shape different*). Data from Experiments 1 and 2 were combined to increase power and because the statistical results were largely the same (see Appendix Tables 5 and 6 results by experiment). A 2 (Shape: Same, Different) × 3 (Transition: Repeat, Baseline, Switch) repeated-measures ANOVA was conducted on recall of probes on the color singleton in the probe task. Results are plotted in Fig. 6.

The ANOVA revealed that only the main effect of transition was statistically significant [*F*(2,60) = 20.42, *MSE* = 0.0049, *p* <.001, η_p_^2^ =.40], reflecting greater recall of probes on repeat trials (89.76%) than on the baseline trials (86.94%) and switch trials (81.81%). The effect of shape and, critically, the interaction were not significant [*F*s < 1, *p*s >.525]. The probe results suggest that the priming effects on the probe task were not due to episodic retrieval. If they were, greater recall should have been observed for the same shape condition than the different shape condition.

To examine episodic retrieval in the search task, a similar 2 (Shape/Response: Same, Different) × 3 (Transition: Repeat, Baseline, Switch) repeated-measures ANOVA was performed on search task RTs, which revealed a main effect of transition [*F*(2,60) = 109.44, *MSE* = 659.26, *p* <.001, η_p_^2^ =.78] that interacted with shape/response [*F*(2,60) = 10.22, *MSE* = 239.48, *p* <.001, η_p_^2^ =.25]. PoP was larger when the target shape/response was the same on Trial *N* as Trial *N* – 1 [76 ms; *t*(30) = 11.91, *SE* = 6.60, *p* <.001, *d* = 1.16] than when the shape/response was different [57 ms; *t*(30) = 10.27, *SE* = 5.57, *p* <.001, *d* = 0.75]. Thus, shape/response had a carryover effect on visual search performance, but did not have a carryover effect on probe responses.

It may also be the case that on probe trials the color singleton’s shape facilitated recall of letters appearing on the non-singleton items that matched the target’s shape. On each trial, two non-singleton items matched the shape of the target (*compatible*) and the other two mismatched (*incompatible*). Probe recall of letters appearing on non-targets was examined based on whether a non-target was compatible or incompatible with the target’s shape. The combined data from Experiments 1 and 2 revealed no difference in recall of probes appearing on non-singletons with shapes that were compatible with the target shape (*M* = 9.02%, *SD* = 7.31%) or incompatible with the target shape [*M* = 8.81%, *SD* = 7.35%; *t*(30) = 0.46, *SE* = 0.0046, *p* =.649, *d* =.08]. In short, these analyses suggest that the shape of the color singleton had no noticeable effect on probe recall, and that the priming effects on probe recall did not reflect episodic retrieval.

## General discussion

The present study used a probe detection task to examine how target activation and distractor inhibition contributed to attentional decision bias in popout search. As discussed earlier, several processes may contribute to selection history effects: (1) pre-attentive gain, (2) bias in attentional decisions, and (3) post-selection retrieval. Because standard PoP tasks may conflate these processes, the random interspersion of the letter probe task with the visual search task allowed a way to isolate attentional bias. Specifically, the quick onset of letters and switch to the probe task should preclude the influence of post-selection retrieval, and selection history should have no influence on the salience of the homogeneous non-targets so any increase in probe recall from non-targets should reflect an attention bias to select the most recent target’s color.

Both Experiment 1 and Experiment 2 revealed PoP in RTs and recall of probes appearing on the color singleton target and the non-target distractors. Specifically, (1) RTs were lower on repeat trials than on baseline or switch trials, (2) recall of probes on the singleton target was greater for repeat trials than baseline or switch trials, and most interestingly (3) recall of probes on the non-target distractors was *greater* on switch trials than baseline or repeat trials, which likely reflected an attentional bias to select the preceding target color. Additionally, the lower percentage of probes recalled from singleton targets on switch trials likely reflects an attentional bias to ignore or suppress the preceding distractor color. In short, the results suggest attentional decision bias was influenced by feature repetition and feature switching.

Experiment 2 assigned two of four possible colors to singleton targets and non-target distractors to examine unique contributions of target activation and distractor inhibition on attentional bias. By creating between trial transitions where only the target’s color, only the non-target colors, or both colors repeated, switched, or were new, Experiment 2 showed that target activation and distractor inhibition contributed to selection history effects in a largely additive manner. Thus, on a given trial, the simultaneous selection of a target’s features and ignoring the distractor’s features biased future attentional decisions toward the previously selected color and away from the previously ignored color.

The two experiments are novel in that they are the first, to my knowledge, to utilize a probe recall task in a popout search task, which has previously been used to study attentional capture effects (Gaspelin et al. 2015) and contingent attentional capture (Burnham, 2020). The present study contributes to the selection history literature by investigating an underlying component to selection history, in this case, how selection history induces an attentional bias to select the features of the most recently attended target as well as ignore the most recent distractors. Previous studies varied the task or response requirement to examine the influence of post-selection processes (e.g., Huang & Pashler, 2005; Thomson & Milliken, 2011, 2013), the delay before a color switch in a display to isolate early activation (e.g., Lamy et al., 2010), or used diffusion modelling to estimate the contributions of selection history processes (e.g., Burnham, 2018a, 2018b, 2023; Tseng et al., 2014). Each of those studies provided evidence that an isolated process or more than one process contributed to the selection history. Additionally, Becker et al. (2023) used eye tracking to examine where first fixations fell and where the eyes dwelled (i.e., targets vs. distractors) following repeat and switch transitions, and indeed found that activation from the previous target biased following eye movements. As most accounts agree that more than one process contributes to selection history effects (Kristjánsson & Campana, 2010), what is needed is a thorough investigation and review of the field to address when, how, and by what order these components engage and contribute to selection history.

Several lingering questions remain. The first is to what extent does performance (priming) in the probe task truly reflect attentional selection and not episodic retrieval. The shape/response similarity analyses did not find any influence of the preceding search target’s response on probe performance, and the probe task has been shown to reflect attentional selection; hence, it is unlikely but not impossible that episodic retrieval continued to the priming effects on probe recall. Second, what is the time course of target activation and distractor inhibition? It has been known for some time that PoP effects arise from displays earlier than Trial *N* – 1. For example, Maljkovic and Nakayama (1994; see also Kristjánsson et al., 2002) found priming from target and non-target features as early as *N* – 5 trials earlier that increased the more recent the priming features appeared. Unfortunately, the preset study was not set up to examine such buildup, as one would need to independently vary the recency of target and distractor color repetition across multiple lags to ensure enough trials.

In conclusion, the present study randomly interleaved a probe detection and recall task with a standard popout search task to examine how attentional decision bias contributes to selection history effects. The results confirm that selection history effects induce an attentional bias through target feature activation and distractor feature inhibition.

## Data Availability

The data will not be deposited; however, all data and experimental (E-Prime) material are available upon request from the author.

## Appendix A

Tables 5 and 6

**Table 5:** Analyses with color singleton shape similarity between Trial *N* – 1 and Trial *N*. Results from separate 2 Color Singleton Shape (Same, Different) × 3 Transition (Repeat, Baseline, Switch) repeated-measures ANOVAs on letter probes recalled from color singletons in Experiments 1 and 2

**Exp.**	**Effect**	***F***	**(** ***df*** **)**	***MSE***	***p***	**η** ^**2**^_**P**_
1	Shape	0.66	1,16	0.003	.428	.04
	Transition	18.94	2,32	0.004	<.001	.54
	Shape x Transition	0.61	2,32	0.006	.547	.04
2	Shape	0.04	1,13	0.006	.846	.00
	Transition	6.78	2,26	0.005	.004	.34
	Shape x Transition	1.72	2,26	0.008	.199	.12

**Table 6:** Analyses with color singleton target response similarity between Trial *N* – 1 and Trial *N*. Results from separate 2 Color Singleton Shape (Same, Different) × 3 Transition (Repeat, Baseline, Switch) repeated-measures ANOVAs on mean response times in the search task in Experiments 1 and 2

**Exp.**	**Effect**	***F***	**(** ***df*** **)**	***MSE***	***p***	**η** ^**2**^_**P**_
1	Response	4.13	1,16	665.60	.059	.20
	Transition	97.72	2,32	557.48	<.001	.86
	Response x Transition	9.11	2,32	227.77	<.001	.36
2	Response	0.07	1,13	1536.51	.795	.00
	Transition	53.93	2,26	426.26	<.001	.80
	Response x Transition	2.36	2,26	254.72	.114	.15

## Footnotes

1 During initial development of the experiment, several research assistants and I found that probe identification was extremely easy with the standard set size of three diamonds/letters (being well within the capacity of short-term memory), which is why set size was increased to five: to avoid ceiling effects.

2 I thank an anonymous reviewer for suggesting that the shape/response similarity between Trial *N* – 1 and Trial *N* targets may lead to episodic retrieval and contribute to PoP and for suggesting the analyses. The analyses focused on the three transitions that were common to both experiments. Analyses of each individual experiment yielded similar patterns (see Appendix Table 5 and 6), and analyses with *experiment* as a between subjects’ factor did not produce interactions with shape similarity.

## Code availability

All code related to the data and experiment will be made available upon request from the author.

## Declarations

### Open Practices Statement

None of the experiments reported in this study were preregistered; however, data or materials used to run the experiments or conduct the analyses will be made available upon request.
